# Operationalizing the Construct of the Internal Saboteur: Development and Psychometric Validation of the Internal Saboteur Scale (ISS)

**DOI:** 10.3390/ejihpe16060080

**Published:** 2026-06-05

**Authors:** Vincenzo Caretti, Eleonora Topino, Andrea Fontana, Gianluigi Di Cesare, Clara Mucci, Adriano Schimmenti, Alessio Gori

**Affiliations:** 1 Department of Human Sciences, LUMSA University, Via della Traspontina, 21, 00193 Rome, Italy; vincenzocaretti@gmail.com (V.C.); a.fontana2@lumsa.it (A.F.); 2 Department of Theoretical and Applied Sciences, eCampus University, Via Isimbardi, 10, 22060 Novedrate, Italy; eleonora.topino@gmail.com; 3 Prevention and Early Intervention Complex Operative Unit, Department of Mental Health, ASL Roma 1, 00193 Rome, Italy; gianluigi.dicesare@aslroma1.it; 4 Department of Human and Social Sciences, University of Bergamo, Via Salvecchio 19, 24129 Bergamo, Italy; clara.mucci@unibg.it; 5 Department of Human and Social Sciences, UKE-Kore University of Enna, Piazza dell’Università, 94100 Enna, Italy; adriano.schimmenti@gmail.com; 6 Department of Health Sciences, University of Florence, Via di San Salvi 12, Pad. 26, 50135 Florence, Italy; 7 Integrated Psychodynamic Psychotherapy Institute (IPPI), Via Ricasoli 32, 50122 Florence, Italy

**Keywords:** internal saboteur, maladaptive inner processes, self-devaluation, rumination, relational expectations, scale validation

## Abstract

The internal saboteur may be understood as a multidimensional configuration of maladaptive inner processes involving recurrent negative self-evaluation, distressing relational expectations, repetitive negative thinking, and self-undermining inner experiences. Within this framework, the present study aimed to develop and examine the psychometric properties of the Internal Saboteur Scale (ISS), a self-report measure designed to assess this construct. A sample of 328 Italian adults (women 71.6%; Mage = 37.37, SD = 14.88) completed the survey. Confirmatory factor analyses supported both an eight-factor correlational model and a theoretically meaningful higher-order model, in which the lower-order dimensions were grouped into four broader domains: Negative Relational Expectations (Expected Rejection; Expected Judgment), Self-Devaluation (Negative Self-Appraisal; Interpersonal Unworthiness), Rumination (Retrospective Rumination; Anticipatory Rumination), and Internal Destructiveness (Helplessness; Defensive Relational Withdrawal). Measurement invariance across gender was also supported. All dimensions showed satisfactory-to-good internal consistency. Furthermore, ISS scores were negatively associated with secure attachment, self-reassurance, and mentalizing and positively associated with insecure attachment, self-criticism, shame, and anger. Overall, the ISS appears to be a theoretically grounded and psychometrically promising instrument for the assessment of maladaptive inner dialogue and self-sabotaging internal processes. It may represent a useful tool for both research and clinical practice, particularly in supporting transdiagnostic assessment and case formulation.

## 1. Introduction

### 1.1. The Internal Saboteur

Recent psychopathology research has increasingly moved beyond strictly categorical formulations and has instead emphasized transdiagnostic processes that cut across diagnostic boundaries (Romer et al., 2024; Cuthbert, 2022). Within this shift, disturbances of the self have gained particular relevance. A recent systematic review showed that self-related dysfunction represents a meaningful transdiagnostic marker across depression and anxiety, suggesting that alterations in how individuals process and represent the self are not peripheral but central to psychopathology (Sabbah & Northoff, 2025). Developmental evidence points in the same direction: maladaptive self-referential processing appears not only to precede increases in depressive symptoms but also to be reinforced by them over time, indicating a potentially reciprocal process of psychological vulnerability (Liu & Tan, 2025). At a broader structural level, research on personality functioning has shown that impairments involving identity, self-direction, and interpersonal relatedness account for substantial transdiagnostic variance and predict later psychosocial impairment beyond narrower symptom dimensions (Kerber et al., 2024).

Within this background, the internal saboteur may be regarded as a particularly promising construct for organizing a set of clinically relevant processes that the literature has often addressed only in partial or adjacent ways. According to Fairbairn (1952), the phenomenon was conceptualized as an internal hostile organization (deriving from a rejecting object generating an anti-libidinal ego in his psychoanalytic terms) that turns against vulnerable, dependent, or needful aspects of the self (Bliss, 2010; Larkins, 2023). In more recent empirical and clinical literature, related experiences have more often been approached through constructs such as self-criticism or the inner critical voice, typically described as a harsh, attacking, and contemptuous stance toward the self that contributes to the maintenance of psychopathological distress (Werner-Riccetti et al., 2025). Other contributions have instead framed similar phenomena in more behavioral terms, defining self-sabotage as self-defeating actions that interfere with health, treatment, or personally valued goals (Sansone et al., 2008). Although each of these formulations captures an important aspect of the phenomenon, each remains only partial. What is still lacking is a broader, multidimensional, and integrated definition capable of linking these manifestations within a single construct that may be meaningfully applied in transdiagnostic clinical practice.

To address this theoretical gap, Caretti et al. (2026) recently proposed that the internal saboteur should be understood as a configuration of dysfunctional self-referential, affective, cognitive, and relational processes that undermine agency, relatedness, and adaptive self-regulation across diagnostic categories.

### 1.2. Core Dimensions of the Internal Saboteur

The internal saboteur reflects the convergence of multiple interrelated psychological processes highlighted in the scientific literature. Within this framework, self-criticism represents a particularly robust component, consistently described as a transdiagnostic process associated with psychological suffering and characterized by self-attack, harsh self-judgment, contempt, and persistent feelings of defectiveness or inadequacy (Zaccari et al., 2024). This process also appears to fluctuate meaningfully in everyday life, intensifying under conditions of fatigue, distress, and perceived criticism, and is associated with heightened negative affect and reduced positive affect (Veilleux et al., 2024), while longitudinal evidence suggests that elevated self-criticism is linked to poorer mental health over time (Wong et al., 2025).

Another central dimension concerns repetitive negative thinking, as rumination and worry are increasingly understood as related expressions of a broader cognitive style marked by repetitive, negatively valenced, and difficult-to-disengage thought (Hitchcock & Frank, 2024); importantly, reductions in these processes have been associated with broader symptom improvement, and interventions directly targeting repetitive negative thinking may be especially effective (Egan et al., 2024; Stenzel et al., 2025).

Maladaptive inner dialogue also extends to the interpersonal domain, taking the form of persistent expectations of rejection, criticism, or negative evaluation, in line with literature on rejection sensitivity and fear of evaluation (Topel et al., 2024), with fear of negative evaluation remaining particularly central because of its associations with lower self-esteem, loneliness, social isolation, and heightened rejection sensitivity (Baldanzini et al., 2025; Gao et al., 2025; Hofmann, 2025).

Finally, this internal configuration may also include a defensive relational tendency, as attachment insecurity has been linked to withdrawal, reduced emotional engagement, and deactivating strategies in close relationships, findings that are coherent with broader models emphasizing impairments in self and interpersonal functioning as clinically meaningful indicators of maladaptation (Chopik et al., 2024; Körner et al., 2025; Walker & Kunst, 2025).

### 1.3. The Present Research

From a clinical perspective, the relevance of assessing the internal saboteur lies in the fact that many maladaptive processes are organized at the level of inner experience before becoming clearly visible in overt behavior or symptom presentation. Existing instruments assess important but circumscribed portions of this domain, including self-criticism and self-reassurance (Gilbert et al., 2004), repetitive negative thinking as a transdiagnostic process (Ehring & Watkins, 2008), and adult attachment patterns relevant to closeness, dependence, and avoidance (Bartholomew & Horowitz, 1991). Some of the central processes regarding the internal saboteur may partially overlap with constructs assessed by symptom measures, including depressive symptom inventories, particularly with regard to self-devaluation, rumination, and helplessness. However, within the ISS, these dimensions are not conceptualized as indicators of a specific symptom syndrome, but as components of a broader and more multifaceted configuration of maladaptive inner dialogue and recurrent self-undermining internal experiences. Accordingly, although adjacent instruments capture relevant aspects of this domain, they were not designed to assess the convergence of these processes within a single multidimensional construct. In the scale-development literature, the construction of a new measure is warranted when adjacent instruments do not adequately represent the full conceptual breadth of the target phenomenon (Boateng et al., 2018; Netemeyer et al., 2003). On this basis, the present study aimed to examine the factorial structure, measurement invariance across gender, internal consistency, and psychometric properties of the Internal Saboteur Scale (ISS). More specifically, the ISS was developed to operationalize the internal saboteur as a multidimensional construct and to provide a clinically meaningful tool for transdiagnostic assessment and case formulation.

## 2. Materials and Methods

### 2.1. Participants, Procedure and Ethics

The sample comprised 328 participants (see Table 1). Mean age was 37.37 years (*SD* = 14.88; range = 18–86). Most participants were women (71.6%, *n* = 235). With regard to marital status, over half of the sample was single (53.0%, *n* = 174), followed by cohabiting (22.9%, *n* = 75) and married participants (19.2%, *n* = 63). In terms of education, the most represented categories were high school diploma (30.8%, *n* = 101) and master’s degree (29.6%, *n* = 97). Regarding occupational status, most participants were employees (48.2%, *n* = 158), followed by students (16.5%, *n* = 54), working students (11.3%, *n* = 37), and freelancers (8.8%, *n* = 29). Participants were recruited online using a snowball sampling strategy and completed an anonymous self-report survey administered through Google Forms. Eligibility criteria required participants to be at least 18 years old, sufficiently proficient in Italian, and willing to provide informed consent.

The survey included questions on demographic characteristics (i.e., sex, age, marital status, occupation, and educational level) as well as the study measures. Informed consent was obtained electronically before participation. The study protocol was approved by the Institutional Ethics Committee of one of the authors’ affiliated institutions.

### 2.2. Development of the Internal Saboteur Scale (ISS)

The Internal Saboteur Scale (ISS) was developed to assess recurrent self-undermining internal experiences, conceptualized as critical inner dialogue, maladaptive self-evaluations, repetitive negative ideation, and distressing expectations concerning interpersonal relationships (Caretti et al., 2026). The ISS was developed to provide a clinically meaningful and psychometrically grounded assessment of maladaptive inner dialogue as a multidimensional construct. In line with this aim, it was conceived as a self-report instrument designed to capture a theoretically coherent but internally differentiated latent structure through items phrased in direct, experience-near language (Boateng et al., 2018). More specifically, the ISS was organized around four higher-order domains, each consisting of two subdomains.

1. Negative Relational Expectations concerns the tendency to expect relational exclusion and negative social evaluation, both of which have been associated with impaired social functioning and heightened sensitivity to interpersonal threat (Topel et al., 2024; Coughlan-Hopkins & Martinelli, 2025). This higher-order factor includes Expected Rejection and Expected Judgment.
  - Expected Rejection refers to the anxious anticipation of being excluded, abandoned, replaced, or refused in emotionally meaningful relationships, coherently with recent conceptualizations of rejection sensitivity emphasizing expectancy, perception, and intense reactivity to rejection cues (Coughlan-Hopkins & Martinelli, 2025).
  - Expected Judgment refers to the anticipation of being criticized, ridiculed, or negatively evaluated for one’s weaknesses, defects, or vulnerabilities, and is thus more closely aligned with recent work on fear of negative evaluation and evaluative threat in social functioning (Fredrick & Luebbe, 2024; Topel et al., 2024; Weeks et al., 2024; Hofmann, 2025).
2. Self-Devaluation concerns a persistently negative and punitive stance toward the self, which recent literature continues to describe as a multifaceted process involving inadequacy, self-contempt, self-dislike, and difficulties in self-reassurance rather than a unitary negative self-view (Zaccari et al., 2024; Veilleux et al., 2024; Wong et al., 2025). This higher-order factor includes Negative Self-Appraisal and Interpersonal Unworthiness. The distinction is also conceptually consistent with the DSM-5-TR Alternative Model for Personality Disorders (American Psychiatric Association, 2022), which organizes severity in personality pathology around impairments in self and interpersonal functioning; in this sense, the present factor differentiates between devaluation primarily directed toward the self and devaluation of the self in relation to others (García et al., 2024; Sharp et al., 2025).
  - Negative Self-Appraisal refers to harsh self-evaluations centered on defectiveness, inadequacy, shame, and self-dislike (Veilleux et al., 2024; Zaccari et al., 2024; Wong et al., 2025).
  - Interpersonal Unworthiness refers to the belief that one is undeserving of affection, care, or relational value, or that one’s presence may represent a burden to others, thereby shifting self-devaluation into the explicitly interpersonal domain (American Psychiatric Association, 2022; García et al., 2024; Sharp et al., 2025; Wong et al., 2025).
3. Rumination captures repetitive negative thinking as a transdiagnostic process that encompasses both dwelling on past adverse experiences and negatively biased anticipation of future threat or failure (Hitchcock & Frank, 2024; Egan et al., 2024; Puccetti et al., 2025). This higher-order factor includes Retrospective Rumination and Anticipatory Rumination.
  - Retrospective Rumination refers to repetitive dwelling on past mistakes, humiliations, losses, or experiences of victimization, with difficulty disengaging from negatively valenced memories (Egan et al., 2024; Puccetti et al., 2025).
  - Anticipatory Rumination, by contrast, refers to repetitive future-oriented negative ideation focused on possible failure, disappointment, deprivation, or threat; in this sense, it overlaps with contemporary accounts of worry and perseverative cognition as future-focused forms of repetitive negative thinking (Hitchcock & Frank, 2024; Egan et al., 2024).
4. Internal Destructiveness represents a more hostile and inhibiting inner stance, marked by internal experiences of futility, uncontrollability, distrust, and defensive withdrawal, which are consistent with both helplessness formulations and attachment-based accounts of deactivating relational strategies (Tafet & Ortiz Alonso, 2025; Chopik et al., 2024; Körner et al., 2025). This higher-order factor includes Helplessness and Defensive Relational Withdrawal.
  - Helplessness refers to internal experiences of futility, blocked agency, and perceived uncontrollability, whereby the individual expects that personal efforts will not meaningfully alter outcomes (Tafet & Ortiz Alonso, 2025).
  - Defensive Relational Withdrawal, instead, refers to a protective but maladaptive tendency to distance oneself from closeness, dependence, and trust because relationships are implicitly experienced as unsafe, unreliable, or threatening; accordingly, it aligns with recent work linking attachment avoidance to emotional distancing, reduced reliance on close others, and withdrawal in intimate relationships (Chopik et al., 2024; Körner et al., 2025).

The development of the ISS items followed a theory-driven and multi-step process, consistent with established recommendations for scale construction and validation in psychological research (Netemeyer et al., 2003). Item generation was grounded in a preliminary review of the literature on the main psychological processes theoretically related to the internal saboteur construct. In line with current methodological recommendations, this phase aimed to ensure conceptual clarity, adequate construct coverage, and close correspondence between the theoretical definition of the construct and its empirical indicators. On the basis of this conceptual framework, a set of items was formulated to represent eight lower-order domains that were expected to cluster into four broader dimensions: Expected Rejection, Expected Judgment, Negative Self-Appraisal, Interpersonal Unworthiness, Retrospective Rumination, Anticipatory Rumination, Helplessness, and Defensive Relational Withdrawal. Special attention was devoted to writing items that referred to subjective and recurrent internal experiences rather than to overt behaviors. For this reason, the statements were formulated in direct and phenomenologically accessible language, so as to capture recurring thoughts, inner voices, self-judgments, and ruminative mental sequences that may emerge spontaneously in everyday life. During item construction, particular care was also taken to maximize semantic clarity, reduce ambiguity, avoid excessive conceptual overlap, and preserve content representativeness across the different facets of the construct. The elaborated version of the ISS consists of 24 items, with three items for each lower-order dimension. Respondents are asked to rate how frequently each internal experience occurred during the previous four weeks using a 5-point Likert scale ranging from 1 (“*not at all*”) to 5 (“*very much*”). The final structure allows the computation of a total ISS score, as well as scores for the four higher-order dimensions and the eight lower-order factors.

### 2.3. Measures

#### 2.3.1. The Internal Saboteur Scale (ISS)

The ISS is a 24-item self-report questionnaire designed to assess maladaptive inner dialogue and recurrent self-sabotaging internal experiences (see Appendix A and Appendix B). The ISS is structured into four higher-order factors, each of which comprises two lower-order dimensions: Negative Relational Expectations (comprising Expected Rejection and Expected Judgment), Self-Devaluation (comprising Negative Self-Appraisal and Interpersonal Unworthiness), Rumination (comprising Retrospective Rumination and Anticipatory Rumination), and Internal Destructiveness (comprising Helplessness and Defensive Relational Withdrawal). Items are rated on a 5-point Likert scale ranging from 1 = *Not at all* to 5 = *Very much*. Subscale scores can be computed by summing the three items corresponding to each lower-order dimension, higher-order factor scores by summing the six relevant items, and a total ISS score by summing all 24 items, with higher scores indicating greater internal sabotaging tendencies.

#### 2.3.2. The Relationship Questionnaire (RQ)

The RQ (Bartholomew & Horowitz, 1991; Carli, 1995) is a 4-item self-report measure of adult attachment based on Bartholomew’s four-category model. In its commonly used format, it includes four short descriptions corresponding to the secure, preoccupied, dismissing, and fearful attachment patterns, and respondents indicate the extent to which each profile matches their typical way of relating to others on a 7-point Likert scale, from 1 (*does not describe me at all*) to 7 (*very much describes me*). Because these attachment scales are single-item, traditional internal consistency coefficients are not considered appropriate for this measure.

#### 2.3.3. The Forms of Self-Criticizing/Attacking and Self-Reassuring Scale (FSCRS)

The FSCRS (Gilbert et al., 2004; Petrocchi & Couyoumdjian, 2016) is a 22-item self-report questionnaire designed to assess how individuals relate to themselves when things go wrong. The scale is composed of three subscales: Inadequate Self, which reflects feelings of personal inadequacy and disappointment with the self; Hated Self, which captures more hostile and self-attacking responses characterized by disgust, contempt, or a desire to hurt aspects of the self; and Reassured Self, which assesses the ability to respond to setbacks with encouragement, warmth, and self-support. Items are rated on a 5-point Likert scale ranging from 0 (*not at all like me*) to 4 (*extremely like me*). In the present sample, internal consistency was good to excellent for all three subscales: Inadequate Self (*α* = 0.912), Hated Self (*α* = 0.832), and Reassured Self (*α* = 0.866).

#### 2.3.4. The Multidimensional Mentalizing Questionnaire (MMQ)

The MMQ (Gori et al., 2021; Gori & Topino, 2023) is a 33-item self-report measure developed to assess mentalizing. The instrument was designed to capture the main components of mentalizing across multiple axes, including self–other, cognitive–affective, inside–outside, and explicit–implicit processes. Items are rated on a 5-point Likert scale ranging from 1 (*Not at all*) to 5 (*A great deal*). An overall MMQ score can be computed, with higher scores indicating better mentalizing ability. In the present sample, internal consistency for the total score was good (*α* = 0.872).

#### 2.3.5. External and Internal Shame Scale (EISS)

The EISS (Ferreira et al., 2022) is a brief 8-item self-report measure designed to assess two related dimensions of shame: External Shame and Internal Shame. External Shame refers to the perception of being negatively evaluated, criticized, devalued, or seen as inadequate by others, whereas Internal Shame reflects a negative and devalued view of the self, including feelings of inferiority, unworthiness, isolation, and self-criticism. Items are rated on a 5-point Likert scale ranging from 0 (*never*) to 4 (*always*). Higher scores indicate greater shame. In the present sample, internal consistency was good for External Shame (*α* = 0.830) and satisfactory for Internal Shame (*α* = 0.748).

#### 2.3.6. The Dimensions of Anger Reactions-5 (DAR-5)

The DAR-5 (Forbes et al., 2014a, 2014b) is a brief 5-item self-report measure designed to assess problematic anger. The scale evaluates five core features of anger experience, namely frequency, intensity, duration, aggressive impulses, and interference with social relationships. Items are rated on a 5-point Likert scale ranging from 1 (*none or almost none of the time*) to 5 (*all or almost all of the time*). Item scores are summed to obtain a total score ranging from 5 to 25, with higher scores indicating greater anger-related difficulties; a cut-off score of 12 or higher has often been used to identify clinically relevant levels of problematic anger. In the present sample, internal consistency for the total score was good (*α* = 0.809).

### 2.4. Data Analysis

All statistical analyses were performed using IBM SPSS Statistics 21.0, IBM SPSS AMOS 24.0 and JASP 0.96 (JASP Team, 2026) for Windows. Descriptive statistics were first computed for all ISS items to assess univariate normality. In line with commonly adopted recommendations for structural equation modeling, absolute skewness values lower than 3 and absolute kurtosis values lower than 7 were considered indicative of acceptable departures from normality (Curran et al., 1996; West et al., 1995). The suitability of the correlation matrix for factor analysis was preliminarily evaluated by means of the Kaiser–Meyer–Olkin (KMO) measure of sampling adequacy and Bartlett’s test of sphericity. KMO values of 0.70 or higher, together with a statistically significant Bartlett’s test, were interpreted as evidence that the data were appropriate for latent structure analysis (Bartlett, 1954; Kaiser, 1974). The hypothesized factor structure of the ISS was then tested through a series of confirmatory factor analyses (CFAs) using maximum-likelihood estimation. Model fit was evaluated using: the discrepancy divided by degrees of freedom (χ^2^/df), Incremental Fit Index (IFI), Tucker–Lewis Index (TLI), Comparative Fit Index (CFI), Root Mean Square Error of Approximation (RMSEA), and the Standardized Root Mean Square Residual (SRMR). Following established recommendations, χ^2^/df values below 5 were considered indicative of reasonable fit (Marsh & Hocevar, 1985). In addition, IFI, TLI, and CFI values ≥ 0.90 were considered indicative of acceptable fit, whereas values ≥ 0.95 suggested good fit; RMSEA and SRMR values ≤ 0.08 were considered acceptable, whereas values ≤ 0.06 indicated good fit (Hu & Bentler, 1999; Schermelleh-Engel et al., 2003). To examine the latent organization of the ISS, five CFA models were tested. The theoretically expected model was tested first: (a) a higher-order model in which the eight lower-order factors loaded onto four broader higher-order dimensions. It was then compared with alternative specifications: (b) an eight-factor correlational model including the lower-order ISS factors as correlated factors, (c) a four-factor model including the main ISS dimensions as correlated factors, (d) a general g-factor model in which all ISS items loaded onto a single latent factor, and (e) a single second-order g-factor model in which the eight lower-order factors loaded onto one general Internal Saboteur factor. Competing nested models were compared using chi-square difference tests (Δχ^2^), with a significant Δχ^2^ indicating a statistically significant decrement in fit for the more constrained model relative to the less constrained one (Anderson & Gerbing, 1988). Model comparison was therefore based on both Δχ^2^ and the overall pattern of fit indices, in order to identify the most theoretically and statistically adequate representation of the scale structure. For the nested model showing a statistically significantly better fit in the χ^2^ difference testing, measurement invariance across gender groups was further examined through multigroup confirmatory factor analysis. Following the hierarchical sequence commonly adopted in the measurement invariance literature, increasingly restrictive models were estimated, namely configural invariance (same factorial structure across groups, with no equality constraints), metric invariance (factor loadings constrained to equality), scalar invariance (factor loadings and item intercepts constrained to equality), and, as an additional restrictive step, structural invariance (latent variances, covariances, and means constrained across groups) (Meredith, 1993). Invariance was considered supported when the decrease in Comparative Fit Index was not greater than 0.010 (ΔCFI ≤ 0.010) and the increase in Root Mean Square Error of Approximation was not greater than 0.015 (ΔRMSEA ≤ 0.015; Cheung & Rensvold, 2002; Chen, 2007). This procedure is consistent with the nested-model comparison approach typically recommended in structural equation modeling for evaluating competing measurement models and their cross-group stability (Anderson & Gerbing, 1988). Internal consistency was evaluated using Cronbach’s alpha (Cronbach, 1951) and McDonald’s omega (McDonald, 2013), with coefficients of 0.70 or higher considered indicative of acceptable reliability (Nunnally & Bernstein, 1994). In addition, Pearson’s r coefficients were computed among both the higher-order and lower-order ISS dimensions in order to examine the pattern of associations between the factors. Discriminant validity was further assessed through the heterotrait–monotrait ratio of correlations (HTMT), calculated using an AMOS plugin (Gaskin & James, 2019). In interpreting the HTMT, values below 0.90 were deemed acceptable (Henseler et al., 2015). Finally, Pearson’s correlation coefficients were computed to examine convergent validity.

## 3. Results

Item analysis for ISS showed that skewness values ranged from +0.420 (ISS5) to +1.984 (ISS11), remaining below the conservative cut-off of |3|. Kurtosis values varied between −0.858 (ISS5) and +3.331 (ISS11), remaining below the recommended cut-off of |7|. The Kaiser-Meyer-Olkin (KMO) measure of sampling adequacy was 0.942, exceeding the recommended threshold of 0.70. In addition, Bartlett’s test of sphericity was statistically significant, *χ*^2^(276) = 5249, *p* < 0.001, confirming the suitability of the correlation matrix for factor analysis.

**Figure 1:**
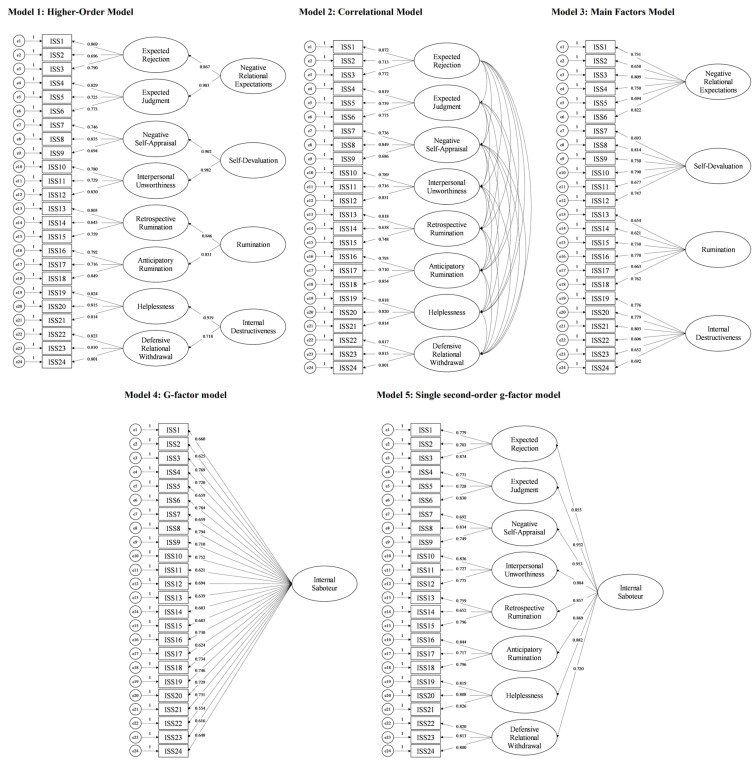
Graphical representation of five tested factor structure models for the ISS.

Concerning the CFA results (see Figure 1 and Table 2), the Correlational Model showed the most favorable fit to the data (*χ*^2^/*df* = 2.54, TLI = 0.917, IFI = 0.933, CFI = 0.932, RMSEA = 0.069, SRMR = 0.043). The Higher-Order Model also showed satisfactory fit indices (*χ*^2^/*df* = 2.80, TLI = 0.903, IFI = 0.917, CFI = 0.917, RMSEA = 0.074, SRMR = 0.051), supporting the theoretical organization of the lower-order factors into broader latent dimensions. The Single Second-Order G-Factor Model showed acceptable values for some indices (*χ*^2^/*df* = 2.87, IFI = 0.911, RMSEA = 0.076, SRMR = 0.050), although its overall fit was less favorable than that of the Correlational and Higher-Order Models. Overall, based on both the pattern of fit indices and the chi-square difference tests (Δχ^2^), the Correlational Model emerged as the best-fitting solution from a statistical perspective, while the Higher-Order Model was retained as theoretically meaningful.

Multigroup CFA was conducted to test the measurement invariance of the Correlational Model across gender groups (see Table 3). The configural model showed acceptable fit, χ^2^(448) = 975.047, *p* = 0.001, CFI = 0.900, TLI = 0.876, IFI = 0.902, RMSEA = 0.060, and SRMR = 0.075. The imposition of equality constraints on factor loadings (metric invariance) resulted in minimal changes in fit (ΔCFI = 0.002; ΔRMSEA = 0.000). Likewise, constraining item intercepts (scalar invariance) produced only negligible additional changes (ΔCFI = 0.003; ΔRMSEA = 0.000). At the structural level, the constrained model showed a slightly larger reduction in CFI, but this change remained within the predefined criteria for invariance (ΔCFI = 0.008; ΔRMSEA = 0.000). Overall, the results supported configural, metric, scalar, and structural invariance of the Correlational Model across gender groups.

Concerning internal consistency, the ISS showed promising results (see Table 4). The total score showed excellent reliability, with both Cronbach’s alpha and McDonald’s omega equal to 0.955. Reliability was also good for the higher-order dimensions, with Cronbach’s alpha coefficients ranging from 0.845 to 0.880 and omega coefficients ranging from 0.842 to 0.882. Similarly, reliability was satisfactory-to-good for the lower-order factors, with alpha coefficients ranging from 0.776 to 0.857 and omega coefficients ranging from 0.787 to 0.860. Regarding discriminant validity, all HTMT values remained below 0.90, supporting an acceptable level of discriminant validity for both the broader domains and the more specific ISS dimensions.

Regarding correlations (see Table 5), the ISS scores showed a coherent pattern of associations with the external variables. More specifically, the ISS total score was negatively correlated with secure attachment (*r* = −0.240, *p* < 0.01), self-reassurance (*r* = −0.346, *p* < 0.01), and mentalizing (*r* = −0.290, *p* < 0.01), whereas it was positively correlated with fearful attachment (*r* = 0.402, *p* < 0.01), preoccupied attachment (*r* = 0.431, *p* < 0.01), dismissing attachment (*r* = 0.135, *p* < 0.05), hated self (*r* = 0.684, *p* < 0.01), inadequate self (*r* = 0.806, *p* < 0.01), external shame (*r* = 0.656, *p* < 0.01), internal shame (*r* = 0.714, *p* < 0.01), and anger (*r* = 0.394, *p* < 0.01).

## 4. Discussion

In recent years, growing attention has been devoted to inner processes that cut across diagnostic categories and contribute to psychological distress through persistent negative self-referential activity (Courchesne et al., 2025; Tan et al., 2024). However, to the authors’ knowledge, no currently available instrument has been specifically designed to capture the convergence of these maladaptive inner processes within a single integrated multidimensional framework, despite the potential usefulness of such an assessment for clinical formulation and transdiagnostic evaluation. Therefore, the present study aimed to develop and examine the psychometric properties of the Internal Saboteur Scale (ISS), a new self-report measure designed to operationalize the Internal Saboteur as a multidimensional construct.

The conceptualization of the ISS and the item-generation process were theory-driven and consistent with current recommendations for psychological scale development, which emphasize the importance of a clearly delimited construct, adequate coverage of its core domains, and close correspondence between the theoretical definition of the construct and its empirical indicators (Boateng et al., 2018; Carpenter, 2018; Clark & Watson, 2019). From this perspective, the ISS was developed to capture different but conceptually related maladaptive inner processes, rather than to reduce the Internal Saboteur to a single undifferentiated dimension. More specifically, four higher-order factors were delineated: Negative Relational Expectations (Expected Rejection, Expected Judgment), reflecting maladaptive interpersonal expectations related to rejection sensitivity and evaluative fears (Coughlan-Hopkins & Martinelli, 2025; Fredrick & Luebbe, 2024; Topel et al., 2024); Self-Devaluation (Negative Self-Appraisal, Interpersonal Unworthiness), reflecting hostile and punitive self-evaluative processes closely linked to shame, inadequacy, and negative self-regard (Veilleux et al., 2024; Zaccari et al., 2024; Wong et al., 2025); Rumination (Retrospective Rumination, Anticipatory Rumination), reflecting repetitive negative thinking focused on both past adverse experiences and future threat or failure (Egan et al., 2024; Hitchcock & Frank, 2024; Puccetti et al., 2025); and Internal Destructiveness (Helplessness, Defensive Relational Withdrawal), reflecting futility, reduced perceived control, distrust, and avoidant disengagement from closeness (Chopik et al., 2024; Körner et al., 2025; Tafet & Ortiz Alonso, 2025). In line with this conceptualization, the findings supported the multidimensional organization of the scale. Importantly, multigroup invariance analyses provided additional support for the stability of the Correlational Model across gender groups. Specifically, configural, metric, scalar, and structural invariance were supported. Overall, these findings suggest that the model operates similarly across gender groups, thus strengthening its generalizability. Furthermore, although the correlational model emerged as the statistically best-fitting solution, the higher-order model also showed satisfactory fit, suggesting that the ISS dimensions may be interpreted both as specific facets and as broader domains of a more general latent configuration. This pattern appears theoretically meaningful, as psychometric literature suggests that complex psychological constructs are often better represented by differentiated but hierarchically related dimensions rather than by either a single global factor or a set of unrelated components (Clark & Watson, 2019). Consistent with this interpretation, the ISS showed good internal consistency (Cronbach, 1951; McDonald, 2013), and a good ability to discriminate both higher-order and lower-order factors (Henseler et al., 2015). Taken together, these findings suggest that the ISS assesses a coherent overarching construct while preserving adequate differentiation among its constituent dimensions, thus supporting its usefulness for capturing distinct yet convergent manifestations of maladaptive inner dialogue.

The findings also showed significant and positive associations between the ISS scores and the variables used to assess convergent validity. Regarding attachment, the ISS total score and subscales were negatively associated with secure attachment and positively associated with both fearful and preoccupied attachment. This is in line with the findings of a recent meta-analysis showing that insecure attachment is positively related to shame, a construct that centrally involves self-depreciation, social withdrawal, and heightened sensitivity to external judgment, all processes that conceptually overlap with several ISS dimensions (Jiang et al., 2026). By contrast, dismissing attachment showed only a small positive association with the ISS total score, and not all dimensions were significantly related to it. Particularly noteworthy, Defensive Relational Withdrawal was positively associated with fearful and preoccupied attachment, but not with dismissing attachment. Although this pattern should be interpreted cautiously, it may suggest that the withdrawal assessed by this subscale does not simply reflect the more deactivating, self-sufficient distancing usually associated with dismissing attachment. Rather, it may capture a form of relational withdrawal that remains saturated with attachment-related anxiety, that is, a defensive retreat motivated by anticipated hurt, rejection, or emotional overload. This interpretation is coherent with recent evidence indicating that attachment avoidance is typically associated with reduced emotional engagement and lower supportive involvement, whereas attachment anxiety is more strongly characterized by hypervigilance to relational threat and intense concern about closeness and rejection (Walker & Kunst, 2025; Rossi et al., 2025). The negative association between ISS scores and mentalizing is also consistent with this reading, as attachment-based models increasingly suggest that insecure attachment and impaired mentalization are closely linked and jointly contribute to maladaptive self–other representations and interpersonal dysfunction (Luyten et al., 2024; Rossi et al., 2025). Furthermore, both the ISS total score and its subscales were negatively associated with Reassured Self and positively associated with Hated Self (Self-Attacking) and Inadequate Self (Self-Criticising). Moreover, the ISS dimensions also showed significant positive associations with anger and with both internal and external shame. Overall, this pattern provides further support for the convergent validity of the scale, as it is coherent with literature describing maladaptive inner dialogue as a configuration characterized by low self-soothing and compassionate self-relating, alongside elevated self-attack, harsh self-evaluation, and emotionally hostile forms of self-focus. In particular, recent work has continued to highlight that self-criticism is closely intertwined with shame-related processes, negative self-evaluation, and self-contempt, whereas self-reassurance appears to play a protective role by mitigating the impact of shame-based experiences and promoting a more adaptive form of self-reflection (Bush & Luchner, 2025; ShamsAlam et al., 2025; Wong et al., 2025). The positive associations with Hated Self (Self-Attacking) and Inadequate Self (Self-Criticising) are also theoretically coherent, given that these dimensions reflect two complementary expressions of self-criticism, namely a more hostile and punitive stance toward the self and a more pervasive sense of personal inadequacy (Papa et al., 2024). Finally, the positive associations with anger and shame further reinforce the clinical plausibility of the ISS, as recent evidence suggests that self-critical processes are frequently accompanied by self-directed hostility, rage, and contempt, and are deeply embedded in shame-based forms of psychological suffering (Azevedo et al., 2025; Bırni et al., 2024; Wong et al., 2025).

### Limitations and Suggestions for Future Research

The present study has some limitations that should be acknowledged. First, participants were recruited online through a snowball sampling procedure. Although this strategy was suitable for an initial psychometric investigation, it may limit the generalizability of the findings. Future research should use more systematic or stratified recruitment procedures. Second, the study relied exclusively on self-report measures. Although this choice was consistent with the aim of assessing subjective inner experiences, it may also have introduced shared-method variance and response biases, including social desirability and inaccuracies in introspection. Future studies should adopt a multi-method approach, integrating self-report data with clinician ratings, interview-based assessments, or informant reports, in order to further examine the convergent and criterion validity of the ISS. Moreover, future research should include established symptom measures, such as depression inventories, to examine the incremental validity of the ISS beyond depressive symptom severity. Third, the present investigation was cross-sectional, which prevented any evaluation of the temporal stability and predictive validity of ISS scores, as well as any conclusion about directionality or causal pathways. Future research should therefore adopt longitudinal designs to examine the stability of the construct over time and to test whether the ISS dimensions function as a predictor, mediator, or moderator of clinically relevant outcomes, including depressive symptoms, psychological distress, impairment, and treatment response. Fourth, although measurement invariance across gender was supported, invariance across other demographic characteristics was not examined. This choice was due to the exploratory nature of this first validation study and to the size and distribution of the present sample, which did not allow sufficiently stable and balanced subgroup comparisons for variables such as age, marital status, or education level. Future studies with larger and more demographically balanced samples should examine whether the ISS factor structure is invariant across additional demographic groups. Finally, the ISS was developed and validated in an Italian sample. Although an English translation is provided, its psychometric properties have not yet been tested in English-speaking or cross-cultural samples. Future studies should therefore examine the cross-cultural validity of the ISS, including linguistic adaptation, factorial structure, reliability, and measurement invariance across countries and cultural groups.

## 5. Conclusions

The Internal Saboteur Scale (ISS) is a theoretically grounded and psychometrically promising self-report measure designed to assess maladaptive inner dialogue through a multidimensional framework. More specifically, it captures recurrent self-undermining processes related to negative relational expectations (Expected Rejection, Expected Judgment), self-devaluation (Negative Self-Appraisal, Interpersonal Unworthiness), rumination (Retrospective Rumination, Anticipatory Rumination), and internal destructiveness (Helplessness, Defensive Relational Withdrawal). In this sense, the ISS may offer a useful contribution to the assessment of clinically relevant inner experiences that often remain only partially addressed by existing instruments. In light of the growing interest in transdiagnostic vulnerability processes, the ISS may represent a valuable tool for both research and clinical practice, particularly in supporting case formulation and the identification of maladaptive self-referential and relational patterns.

## Figures and Tables

**Table 1:** Demographic characteristics of the sample (*N* = 328).

*Characteristics*		*M* ± *SD*	*n*	%
	Age (years)	37.37 ± 14.88		
*Gender*				
	Females		235	71.6
	Males		93	28.4
*Marital Status*				
	Single		174	53.0
	Cohabiting		75	22.9
	Married		63	19.2
	Separated		7	2.1
	Divorced		7	2.1
	Widowed		2	0.6
*Education level*				
	Middle school		17	5.2
	High school diploma		101	30.8
	Bachelor’s degree		73	22.3
	Master’s degree		97	29.6
	Postgraduate specialization		40	12.2
*Working condition*				
	Manager		6	1.8
	Student		54	16.5
	Working student		37	11.3
	Employee		158	48.2
	Entrepreneur		10	3.0
	Homemaker		6	1.8
	Retired		16	4.9
	Freelance		29	8.8
	Unemployed		10	3.0
	Shopkeeper		2	0.6

**Table 2:** Fit statistics of the ISS for five CFA models and chi-square difference test.

*Model*	*χ* ^2^	*df*	*p*	*TLI*	*IFI*	*CFI*	*RMSEA*	*SRMR*	*Models* *Comparison*	Δ*χ*^2^	Δ*df*	*p*
Model 1: Higher-Order Model	665.283	238	<0.001	0.903	0.917	0.917	0.074	0.051	—	—	—	—
Model 2: Correlational Model	569.648	224	<0.001	0.917	0.933	0.932	0.069	0.043	M1–M2	95.635	14	<0.001
Model 3: Main Factors Model	1047.429	236	<0.001	0.824	0.844	0.843	0.100	0.060	M3–M1	382.146	2	<0.001
									M3–M2	477.781	12	<0.001
Model 4: G-factor Model	1262.276	252	<0.001	0.784	0.804	0.731	0.111	0.065	M4–M1	596.993	14	<0.001
									M4–M2	692.628	28	<0.001
									M4–M3	214.847	16	<0.001
Model 5: Single second-order g-factor Model	700.197	244	<0.001	0.899	0.911	0.855	0.076	0.050	M5–M1	34.914	6	<0.001
									M5–M2	130.549	20	<0.001
									M5–M3	347.232	8	<0.001
									M5–M4	562.079	8	<0.001

Notes. *χ*^2^ = chi-square value of model fit; *df* = degrees of freedom; TLI = Tucker–Lewis Index; IFI = Incremental Fit Index; CFI = Comparative Fit Index; RMSEA = Root Mean Square Error of Approximation; SRMR = Standardized Root Mean Square Residual. Δ*χ*^2^ = difference in *χ*^2^ values between the compared models; Δ*df* = difference in number of degrees of freedom between the compared models.

**Table 3:** Multigroup analyses to test the measurement invariance for the Correlational Model across gender groups.

	*χ* ^2^	*df*	*p*	*TLI*	*IFI*	*CFI*	*RMSEA*	*SRMR*	Δ*CFI*	Δ*RMSEA*
Configural invariance	975.047	448	0.001	0.876	0.902	0.900	0.060	0.075	-	-
Metric invariance	1000.097	464	0.001	0.879	0.902	0.898	0.060	0.073	0.002	0.000
Scalar invariance	1041.850	488	0.001	0.881	0.896	0.895	0.060	0.073	0.003	0.000
Structural invariance	1116.485	524	0.001	0.881	0.888	0.887	0.060	0.103	0.008	0.000

Notes. *χ*^2^ = chi-square value of model fit; *df* = degrees of freedom; TLI = Tucker–Lewis Index; IFI = Incremental Fit Index; CFI = Comparative Fit Index; RMSEA = Root Mean Square Error of Approximation; SRMR = Standardized Root Mean Square Residual; ΔCFI = difference in CFI between the compared models; ΔRMSEA = difference in RMSEA between the compared models.

**Table 4:** Internal consistency, inter-factor correlations, and HTMT analysis for the ISS.

*Dimensions*		*Items (N)*	*α*	*ω*	*Inter-Factor Correlations (Above the Diagonal) and HTMT Analysis (Below the Diagonal).*							
					1	2	3	4	5	6	7	8
Total Score		24	0.955	0.955	—	—	—	—	—	—	—	—
Higher-order factors												
	1. Negative Relational Expectations	6	0.880	0.882	1	0.779	0.773	0.720	—	—	—	—
	2. Self-Devaluation	6	0.879	0.881	0.878	1	0.761	0.717	—	—	—	—
	3. Rumination	6	0.845	0.842	0.891	0.881	1	0.759	—	—	—	—
	4. Internal Destructiveness	6	0.869	0.870	0.830	0.825	0.890	1	—	—	—	—
Lower-order factors												
	1. Expected Rejection	3	0.830	0.835	1	0.686	0.618	0.604	0.585	0.587	0.623	0.605
	2. Expected Judgment	3	0.822	0.823	0.830	1	0.713	0.714	0.715	0.630	0.610	0.509
	3. Negative Self-Appraisal	3	0.798	0.798	0.760	0.879	1	0.726	0.648	0.655	0.712	0.559
	4. Interpersonal Unworthiness	3	0.818	0.824	0.723	0.854	0.887	1	0.584	0.630	0.617	0.471
	5. Retrospective Rumination	3	0.776	0.787	0.729	0.896	0.823	0.727	1	0.584	0.602	0.556
	6. Anticipatory Rumination	3	0.824	0.827	0.709	0.769	0.811	0.760	0.734	1	0.730	0.509
	7. Helplessness	3	0.857	0.860	0.740	0.728	0.865	0.732	0.738	0.874	1	0.563
	8. Defensive Relational Withdrawal	3	0.848	0.852	0.723	0.611	0.679	0.563	0.684	0.607	0.656	1

Note. *α* = Cronbach’s alpha; *ω* = McDonald’s omega. Correlations are reported above the diagonal and HTMT values below the diagonal. All Pearson inter-factor correlations were significant at *p* < 0.01.

**Table 5:** Pearson’s correlations between variables used to assess convergent validity (vertical axis) and ISS dimensions (horizontal axis).

	ISS Total	F1. Negative Relational Expectations	Expected Rejection	Expected Judgment	F2. Self-Devaluation	Negative Self-Appraisal	Interpersonal Unworthiness	F3. Rumination	Retrospective Rumination	Anticipatory Rumination	F4. Internal Destructiveness	Helplessness	Defensive Relational Withdrawal
Secure Attachment	−0.240 **	−0.213 **	−0.271 **	−0.127 *	−0.209 **	−0.187 **	−0.201 **	−0.156 **	−0.137 *	−0.140 *	−0.290 **	−0.255 **	−0.258 **
Fearful Attachment	0.402 **	0.365 **	0.360 **	0.313 **	0.357 **	0.313 **	0.351 **	0.336 **	0.293 **	0.306 **	0.392 **	0.306 **	0.385 **
Preoccupied Attachment	0.431 **	0.483 **	0.464 **	0.424 **	0.421 **	0.410 **	0.372 **	0.302 **	0.288 **	0.249 **	0.345 **	0.306 **	0.304 **
Dismissing Attachment	0.135 *	0.070	0.051	0.077	0.188 **	0.159 **	0.191 **	0.091	0.070	0.093	0.138 *	0.142 *	0.102
Hated Self (Self-Attacking)	0.684 **	0.609 **	0.552 **	0.566 **	0.638 **	0.637 **	0.548 **	0.616 **	0.541 **	0.556 **	0.606 **	0.611 **	0.463 **
Inadequate Self (Self-Criticising)	0.806 **	0.693 **	0.566 **	0.700 **	0.783 **	0.789 **	0.663 **	0.763 **	0.705 **	0.651 **	0.671 **	0.701 **	0.490 **
Reassured Self (Self-Reassuring)	−0.346 **	−0.283 **	−0.261 **	−0.259 **	−0.348 **	−0.365 **	−0.280 **	−0.319 **	−0.252 **	−0.320 **	−0.298 **	−0.371 **	−0.159 **
Mentalizing	−0.290 **	−0.254 **	−0.274 **	−0.196 **	−0.277 **	−0.252 **	−0.262 **	−0.231 **	−0.181 **	−0.232 **	−0.285 **	−0.285 **	−0.221 **
External Shame	0.656 **	0.655 **	0.565 **	0.635 **	0.604 **	0.546 **	0.577 **	0.562 **	0.510 **	0.489 **	0.545 **	0.502 **	0.462 **
Internal Shame	0.714 **	0.664 **	0.581 **	0.637 **	0.705 **	0.666 **	0.644 **	0.622 **	0.546 **	0.561 **	0.584 **	0.575 **	0.460 **
Anger	0.394 **	0.389 **	0.359 **	0.355 **	0.319 **	0.303 **	0.288 **	0.349 **	0.355 **	0.263 **	0.363 **	0.313 **	0.329 **

Note: Bold values indicate significant correlations. **. Correlation is significant at the 0.01 level (2-tailed). *. Correlation is significant at the 0.05 level (2-tailed).

## Data Availability

Data presented in this study are available on request from the corresponding author.

## Appendix A. Internal Saboteur Scale (ISS)–Italian Version

Le frasi che seguono descrivono pensieri ricorrenti, voci interne e modalità di dialogo interiore che ciascuno può sperimentare rispetto a sé stesso e alle proprie relazioni.

L’attenzione non è quindi rivolta ai comportamenti esterni, ma a ciò che accade dentro la mente, sotto forma di idee, commenti, giudizi o rimuginii che possono emergere spontaneamente nella vita quotidiana. Le chiediamo di indicare con quale frequenza tali pensieri o voci interiori si sono presentati nelle ultime quattro settimane, scegliendo la risposta che meglio rappresenta la sua esperienza.

Utilizzi per favore la seguente scala di risposta:

*Per niente*	*Un po’*	*Abbastanza*	*Molto*	*Moltissimo*
**1**	**2**	**3**	**4**	**5**

1. Sono convinto che nelle amicizie, finirò per essere escluso o sostituito da altri.	1	2	3	4	5
2. Sento sempre di essere trascurato dalle persone a cui sono legato affettivamente.	1	2	3	4	5
3. Penso che nelle relazioni sociali prima o poi sarò rifiutato.	1	2	3	4	5
4. Nella mia mente mi aspetto di essere deriso o preso in giro dagli altri.	1	2	3	4	5
5. Nella mia mente ritorna il pensiero che gli altri mi possano giudicare per le mie debolezze o difetti.	1	2	3	4	5
6. Sento che le persone a cui sono affezionato possano pensare male di me.	1	2	3	4	5
7. Quando ricevo un complimento, dentro di me non sento di meritarlo davvero.	1	2	3	4	5
8. Dentro di me risuona l’idea che non valgo nulla.	1	2	3	4	5
9. Quando mi guardo dentro, provo disagio per aspetti di me che sono inaccettabili.	1	2	3	4	5
10. Dentro di me sento di essere un peso per gli altri.	1	2	3	4	5
11. Penso che non merito affetto da parte degli altri.	1	2	3	4	5
12. Una voce dentro di me continua a dirmi che non sono all’altezza nelle relazioni sociali.	1	2	3	4	5
13. Rivivo mentalmente i miei errori passati come se fossero ancora presenti.	1	2	3	4	5
14. Mi ritrovo a rivivere situazioni in cui in passato sono stato vittima di qualcuno.	1	2	3	4	5
15. Mi ritorna il pensiero di situazioni in cui ho fatto una brutta figura.	1	2	3	4	5
16. Nelle mie fantasie mi ripeto che la mia vita andrà a rotoli.	1	2	3	4	5
17. Rimugino sull’idea che alla fine resterò povero e senza risorse.	1	2	3	4	5
18. Penso costantemente che deluderò le aspettative di chiunque.	1	2	3	4	5
19. Dentro di me una voce insiste che qualunque progetto che faccio è destinato a fallire.	1	2	3	4	5
20. La mia testa è piena di pensieri che mi dicono che qualunque cosa faccia non servirà a nulla.	1	2	3	4	5
21. Una voce dentro di me mi dice che, per quanto mi impegni, non potrò cambiare mai.	1	2	3	4	5
22. Una voce dentro di me insiste che non devo fidarmi di nessuno.	1	2	3	4	5
23. Anche quando una relazione va bene, ho il desiderio di troncarla per paura di soffrire.	1	2	3	4	5
24. Sono convinto che nessuno potrà mai capirmi davvero.	1	2	3	4	5

## Appendix B. Internal Saboteur Scale (ISS)–English Translation

The following statements describe recurring thoughts, inner voices, and forms of inner dialogue that people may experience in relation to themselves and their relationships. The focus is therefore not on outward behaviors, but on what takes place in the mind, in the form of ideas, comments, judgments, or ruminative thoughts that may arise spontaneously in everyday life. Please indicate how often these thoughts or inner voices have occurred during the past four weeks by selecting the response that best reflects your experience.

Please use the following response scale:

*Not at all*	*A little*	*Moderately*	*Very much*	*Extremely*
**1**	**2**	**3**	**4**	**5**

1. I am convinced that, in friendships, I will eventually be excluded or replaced by someone else.	1	2	3	4	5
2. I often feel overlooked by the people I am emotionally close to.	1	2	3	4	5
3. I think that, sooner or later, I will be rejected in social relationships.	1	2	3	4	5
4. In my mind, I expect other people to ridicule me or make fun of me.	1	2	3	4	5
5. I keep thinking that other people may judge me for my weaknesses or flaws.	1	2	3	4	5
6. I feel that the people I care about may think badly of me.	1	2	3	4	5
7. When someone compliments me, deep down I do not really feel I deserve it.	1	2	3	4	5
8. Deep down, I feel that I am worthless.	1	2	3	4	5
9. When I look inside myself, I feel uncomfortable with parts of myself that I find unacceptable.	1	2	3	4	5
10. Deep down, I feel that I am a burden to others.	1	2	3	4	5
11. I feel that I do not deserve affection from others.	1	2	3	4	5
12. A voice inside me keeps telling me that I am not good enough in social relationships.	1	2	3	4	5
13. I mentally relive my past mistakes as if they were still happening.	1	2	3	4	5
14. I find myself reliving situations in which I was hurt or taken advantage of by someone in the past.	1	2	3	4	5
15. Thoughts about situations in which I embarrassed myself keep coming back to me.	1	2	3	4	5
16. In my mind, I keep telling myself that my life will fall apart.	1	2	3	4	5
17. I dwell on the idea that I will eventually end up poor and without resources.	1	2	3	4	5
18. I constantly think that I will disappoint everyone.	1	2	3	4	5
19. A voice inside me insists that any plan I make is bound to fail.	1	2	3	4	5
20. My mind is full of thoughts telling me that nothing I do will make any difference.	1	2	3	4	5
21. A voice inside me tells me that, no matter how hard I try, I will never really change.	1	2	3	4	5
22. A voice inside me keeps telling me that I should not trust anyone.	1	2	3	4	5
23. Even when a relationship is going well, I feel the urge to end it for fear of getting hurt.	1	2	3	4	5
24. I am convinced that no one will ever truly understand me.	1	2	3	4	5
